# Neutrophil Maturation, Reactivity and Granularity Research Parameters to Characterize and Differentiate Convalescent Patients from Active SARS-CoV-2 Infection

**DOI:** 10.3390/cells10092332

**Published:** 2021-09-06

**Authors:** Iwona Kwiecień, Elżbieta Rutkowska, Katarzyna Kulik, Krzysztof Kłos, Katarzyna Plewka, Agata Raniszewska, Piotr Rzepecki, Andrzej Chciałowski

**Affiliations:** 1 Laboratory of Hematology and Flow Cytometry, Department of Internal Medicine and Hematology, Military Institute of Medicine, Szaserów 128, 04-141 Warsaw, Poland; erutkowska@wim.mil.pl (E.R.); kkulik@wim.mil.pl (K.K.); araniszewska@wim.mil.pl (A.R.); 2 Department of Infectious Diseases and Allergology, Military Institute of Medicine, Szaserów 128, 04-141 Warsaw, Poland; kklos@wim.mil.pl (K.K.); kplewka@wim.mil.pl (K.P.); achcialowski@wim.mil.pl (A.C.); 3 Department of Internal Medicine and Hematology, Military Institute of Medicine, Szaserów 128, 04-141 Warsaw, Poland; przepecki@wim.mil.pl

**Keywords:** neutrophils, reactivity of neutrophils, neutrophils granularity index, COVID-19, morphological parameters, SARS-CoV-2

## Abstract

Studying the dynamics changes of neutrophils during innate immune response in coronavirus 2019 (COVID-19) can help understand the pathogenesis of this disease. The aim of the study was to assess the usefulness of new neutrophil activation parameters: Immature Granulocyte (IG), Neutrophil Reactivity Intensity (NEUT-RI), Neutrophil Granularity Intensity (NEUT-GI), and data relating to granularity, activity, and neutrophil volume (NE-WX, NE-WY, NE-WZ) available in hematology analyzers to distinguish convalescent patients from patients with active SARS-CoV-2 infection and healthy controls (HC). The study group consisted of 79 patients with a confirmed positive RT-PCR test for SARS-CoV2 infection, 71 convalescent patients, and 20 HC. We observed leukopenia with neutrophilia in patients with active infection compared to convalescents and HC. The IG median absolute count was higher in convalescent patients than in COVID-19 and HC (respectively, 0.08 vs. 0.03 vs. 0.02, *p* < 0.0001). The value of the NEUT-RI parameter was the highest in HC and the lowest in convalescents (48.3 vs. 43.7, *p* < 0.0001). We observed the highest proportion of NE-WX, NE-WY, and NE-WZ parameters in HC, without differences between the COVID-19 and convalescent groups. New neutrophil parameters can be useful tools to assess neutrophils’ activity and functionalities in the immune response during infection and recovery from COVID-19 disease.

## 1. Introduction

Coronavirus disease 2019 (COVID-19) is a diagnosed pneumonia caused by severe acute respiratory syndrome coronavirus 2 (SARS-CoV-2) that has quickly spread across continents leading to a global pandemic [1]. The course of the disease can vary greatly, from asymptomatic to highly critical, and can rapidly develop into acute respiratory syndrome and may even lead to death [2]. COVID-19 is characterized by clinical symptoms, such as fever, cough, and dyspnea, with chest radiographs showing intrusive lesions in both lungs. The most common morphological and biochemical parameters include lymphopenia, neutrophilia, an elevated neutrophil to lymphocyte ratio (NLR), D-dimer concentrations, level of inflammation marker C Reactive Protein (CRP), lactate dehydrogenase (LDH), or an elevated reactive lymphocyte (RE-LYMP) parameter, in addition to cytokine release syndrome [3,4,5,6].

Ongoing research has shown that lymphocytes and the subsets of CD4+ T cells, CD8+ T cells, B cells, and natural killer (NK) cells also play an important role in the maintenance of immune system function during COVID-19 [7]. COVID-19 patients exhibit a reduction in the absolute number of lymphocytes, including CD4+ and CD8+ T lymphocytes, which display markers related to activation or exhaustion/senescence, in addition to altered expression of master regulators and several chemokine receptors [8,9]. Routine laboratory parameters and the percentage or absolute number of leukocyte and lymphocyte subpopulations may be related to disease severity and prognosis [10,11].

Recently, attention has been paid to the role of neutrophils in immune responses in COVID-19 patients and the possibility of developing new targeted therapies which may help reduce COVID-19 mortality [12,13].

Neutrophils are essential for innate immunity and play an important role in inflammation [14]. The increased level of neutrophils is well documented in COVID-19 patients [15]. It is well known that the neutrophilia are a predictor of poor outcome [16,17], but the role of these cells in the pathophysiology of COVID-19 is not well described. To understand their importance in the development of COVID-19, it is necessary to examine not only their quantity, but also their functionality, degree of activation, and exhaustion. Several studies have reported changes in neutrophils’ activation, and the change in the degranulation of the phenotype indicates their dysfunctionality and the secretion of neutrophil extracellular traps (NETs) [18,19,20,21]. Therefore, there is a need to search for parameters that would allow the assessment of the functional activity of neutrophils in an accessible, simple, and quick manner.

As a result of recent technological advances, hematological analyzers not only allow counting and differentiation of the leukocyte populations, but also provide new additional parameters for determining the degree of activity and maturity of cells. These parameters are available with routine morphology and can provide a wealth of information about neutrophils and their role in the pathogenesis of COVID-19.

The Sysmex XN-Series hematology analyzer (Sysmex Corp., Kobe, Japan) provides a full blood count with novel parameters, which allows differentiating activated inflammatory cells—reactive lymphocytes (RE-LYMP)—and neutrophils—neutrophil reactivity intensity (NEUT-RI) and neutrophil granularity intensity (NEUT-GI). This process is carried out by fluorescence emission based on the combination of side scatter (SSC), forward scatter (FSC), and fluorescence intensity (SFL) of nucleated cells [22,23]. In our previous work, we demonstrated the usability of RE-LYMP in combination with flow cytometric activation markers for identifying and distinguishing COVID-19 in patients from other viruses and healthy donors [5].

Changes in neutrophil activation are seen in COVID-19, but its importance in recovery is unknown [24,25].

The aim of the current study was to assess the selected new neutrophil maturation, reactivity, and granularity parameters to characterize convalescent patients, and the possibility of using these parameters to distinguish them from patients with active SARS-CoV-2 infection and healthy volunteers.

## 2. Materials and Methods

### 2.1. Study Participants

The study group consisted of 79 patients with positive SARS-CoV2 infection, 71 convalescent patients, and 20 healthy controls (HC). Samples were collected from 10 May 2020 to 1 February 2021 at the Military Institute of Medicine (Department of Internal Medicine and Hematology, Laboratory of Hematology and Flow Cytometry and the Department of Infectious Diseases and Allergology).

Peripheral blood (PB) samples were collected from all patients. All evaluated hematological parameters were measured on PB samples collected in EDTA-K3 tubes (Beckton Dickinson, Franklin Lakes, NJ, USA), processed within 2 h from the sample collection on a Sysmex XN-series hematology system (Sysmex Co., Kobe, Japan).

Patients with a SARS-CoV-2 positive test were confirmed by real-time reverse transcriptase-polymerase chain reaction (RT-PCR) assay for nasal and pharyngeal swab specimens according to the WHO guidelines.

Patients with a SARS-CoV-2 positive test were newly admitted (Department of Infectious Diseases and Allergology). COVID-19 patients’ characteristics including age, gender, clinical symptoms, diseases comorbidities, and information about saturation, radiological images, oxygen supplementation, and invasive ventilation are presented in Table 1. The baseline clinical condition on admission was classified as symptomatic unstable with SpO2 at 86% to 98%, and symptomatic unstable with SpO2 ≤ 90% or acute respiratory distress syndrome. Sixty-two patients had images of interstitial densities in the lungs by radiologically. Sixty-four patients required oxygen ventilation and 7 required invasive ventilation.

The decision about the treatment regimen was taken entirely by the treating physician based on the current knowledge and recommendations of the Polish Association of Epidemiologists and Infectiologists [26]. Throughout the analyzed period, low-molecular-weight heparin at prophylactic or therapeutic doses, dexamethasone in patients receiving remdesivir and oxygen therapy or lopinavir/ritonavir applied in the first period of the pandemic, antibiotic therapy in the case of secondary bacterial infection, and oral or intravenous hydration and symptomatic treatment were recommended in patients with respiratory failure in accordance with these national guidelines. Of the analyzed patients, seven patients were treated in the intensive care unit (ICU). There was no co-infection in the analyzed group of patients. One patient died and another one required treatment in the Department of Endovascular Surgery for lower limb artery thrombosis. Three patients had Clostridioides difficile gastrointestinal tract co-infection. The mean time of hospitalization was 15.8 ± 10 days.

The patients were considered convalescent after clinical stabilization: improvement in general condition, body temperature normalization, cough and dyspnea subsided, normalization of inflammatory parameters, and elimination of SARS-CoV-2 virus (two negative results of nasopharyngeal swabs separated by 48 h) with no abnormalities in physical examination, no complaints, and in good general condition. The convalescent group consisted of 71 patients: 23 female and 48 male mean age: 59.3 ± 14.5 years. There were 46 cases in the convalescent group that were the same patients after COVID-19 recovery. Among the remaining 25 convalescents, comorbidities were evident as follows: 3 diabetes, 8 hypertension, 3 obesity, 2 coronary heart disease, and 1 neoplastic disease.

The twenty age-matched healthy individuals were used as the control group: 15 female, 5 male, mean age: 54.9 ± 10.1 years.

The blood samples used in the study were taken during routine diagnostics and were approved by the Ethics Committee of the Military Institute of Medicine, and all patients gave informed consent (Military Institute of Medicine Ethics Committee number: 47/WIM/2020).

### 2.2. Morphological and New Sysmex Parameters Connected with Neutrophils

Measurement of neutrophil activation parameters using the Sysmex XN-1500 analyzer ((Sysmex Corp., Kobe, Japan) is based on the fluorescence flow-cytometry method. Cells are permeabilized and then labeled with a fluorescent dye that binds to the nucleic acids. Cells are then categorized according to the intensity of the FSC light, which indicates the volume of the cells. SSC light provides information about the internal cell structure and granularity and SFL light indicates the amount of DNA and RNA present in the cell [27].

The measurement signals related to SSC and SFL were analyzed and are plotted on a scattergram. The Immature Granulocyte (IG) fraction includes promyelocytes, myelocytes, and metamyelocytes, and is above the neutrophil cluster in the white blood cell (WBC) differential fluorescence (WDF) scattergram [28].

The increase in metabolic activity of neutrophils is accompanied by a higher quantity of nucleic acids that more intensively bind the fluorescent dye, leading to an increase in the SFL.

The NEUT-RI parameter represents the mean value of fluorescence intensity (FI) associated with neutrophil reactivity. In addition, activated neutrophils are characterized by an increase in granularity and the presence of vacuoles, which causes an increase in the SSC value and a change in the position of the neutrophil cloud on the scattergram. This is reflected in the change in the NEUT-GI value expressed as the Scatter Intensity (SI) [29].

The NE-WY parameter is calculated based on the spread around the mean fluorescence value on the neutrophil scattergram. It represents the range of fluorescence distribution for neutrophils, excluding values exceeding below 20% of the peak height of the distribution curve [30]. The NE-WZ and NE-WX parameters are calculated analogously to the NE-WY and correspond to the width of dispersion of neutrophil size and complexity, respectively [24]. All new neutrophil parameters were collected and summarized in the Table 1.

### 2.3. Statistical Analysis

The Statistica 13.0 software (TIBCO Software, Palo Alto, CA, USA) was used for statistical analysis. For group comparison the Mann–Whitney U test, the Kruskal–Wallis ANOVA test, and post-hoc analysis test were used. Relations between the quantitative variables were analyzed by Spearman correlations. A *p*-value < 0.05 was considered to be statistically significant.

## 3. Results

### 3.1. Patients Characteristic with Basic Leukocytes Subpopulation

The characteristics of the COVID-19 positive investigated group are summarized in the Table 2. The routine blood count values, such as WBC, neutrophil, lymphocyte, monocyte, eosinophil, and basophil absolute counts and proportion were compared between COVID-19 patients, convalescent patients, and HC (Table 2). The WBC, lymphocyte, monocyte, eosinophil, and basophil absolute counts were lower in COVID-19 patients than in convalescent patients. The proportion of lymphocytes, monocytes, eosinophils, and basophils were also lower in COVID-19 patients than in convalescent patients. There were higher PLT counts in the convalescent than HC and COVID-19 patients; the PLT counts were significantly different between convalescent and COVID-19 patients, and convalescent patients with COVID-19 and HC.

**Figure 1:**
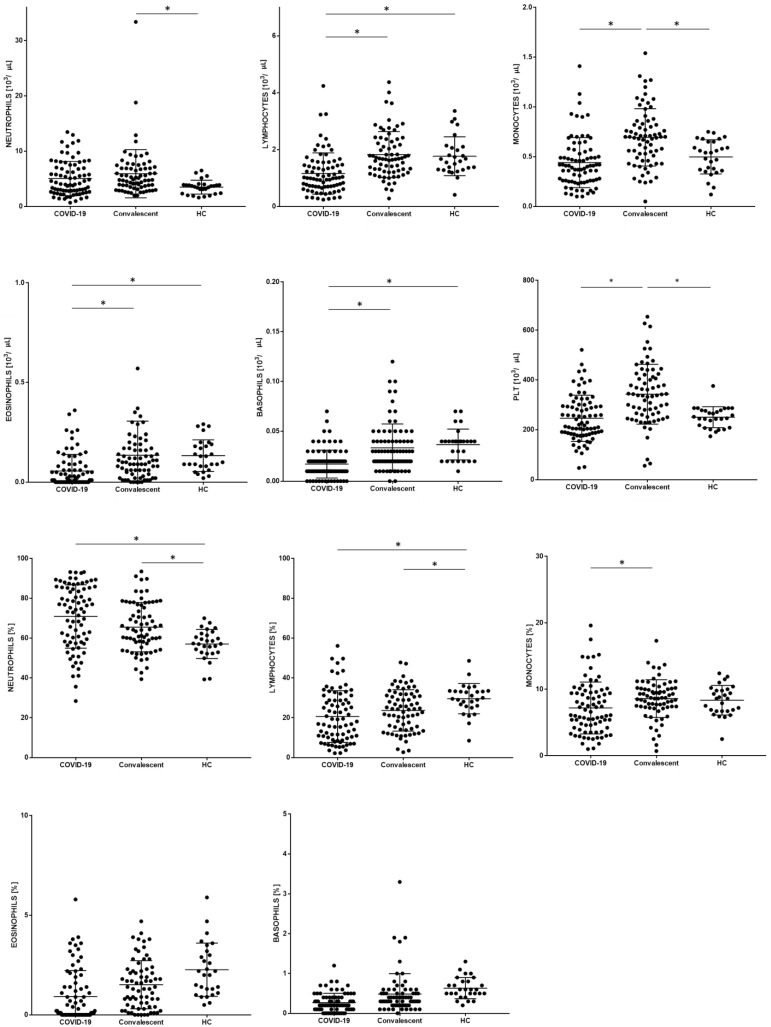
Differences in the proportion of Sysmex morphological parameters between patients with COVID-19, convalescent, and healthy control (HC) groups. Data expressed as median (Min–Max). * indicates *p* statistically significant.

There were higher neutrophil proportions in COVID-19 patients than in convalescent patients and HC; neutrophil proportions were significantly different between COVID-19 patients and HC, and between convalescent patients and HC. The differences in Sysmex morphological parameters between patients with COVID-19, and convalescents with COVID-19 and HC, are presented in Table 3 and Figure 1.

### 3.2. New Neutrophil Parameters

**Figure A1:**
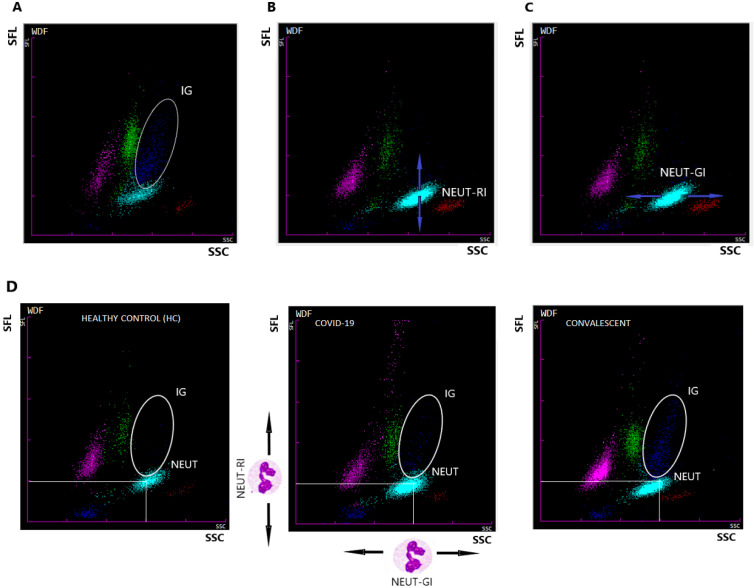
Sample screenshot scattergrams from the Sysmex XN-1500 analysis showing: (**A**) immature granulocytes (IG); (**B**) neutrophil reactivity intensity parameter (NEUT-RI indicated by increased fluorescence intensity, relative shift from blue arrow; (**C**) neutrophil granularity intensity (NEUT-GI), relative shift from blue arrow; (**D**) changes in the position of neutrophil population between exemplary cases of patients from healthy control (HC), COVID-19 group, and convalescent patients. The scattergrams were plotted using side scatter (SSC, intracellular structure) on the *x*-axis and the side fluorescence signal (SFL, presence of nucleic acid) on the *y*-axis. Each dot represents one cell.

Additionally, the study evaluated new Sysmex research parameters connected with maturation, neutrophil reactivity, and granularity (sample scattergram screenshots from the Sysmex XN-1500 analysis software, showing selected parameters—NEUT-RI, NEUT-GI, and IG—are presented in Appendix A: Figure A1). The IG median absolute count was the higher in convalescent patients with COVID-19 than in COVID-19 patients and HC. The IG median proportion was higher in COVID-19 patients than in HC and convalescent patients with COVID-19 than in HC, and no differences were found between convalescent patients with COVID-19 and COVID-19 patients. The proportion of the NEUT-RI parameter was higher in COVID-19 patients than in convalescent patients with COVID-19, and lower in COVID-19 patients, than in HC. The lower proportion of the NEUT-RI parameter was observed in convalescent patients with COVID-19. We did not observe a difference between the NEUT-GI parameters of the study groups.

**Figure 2:**
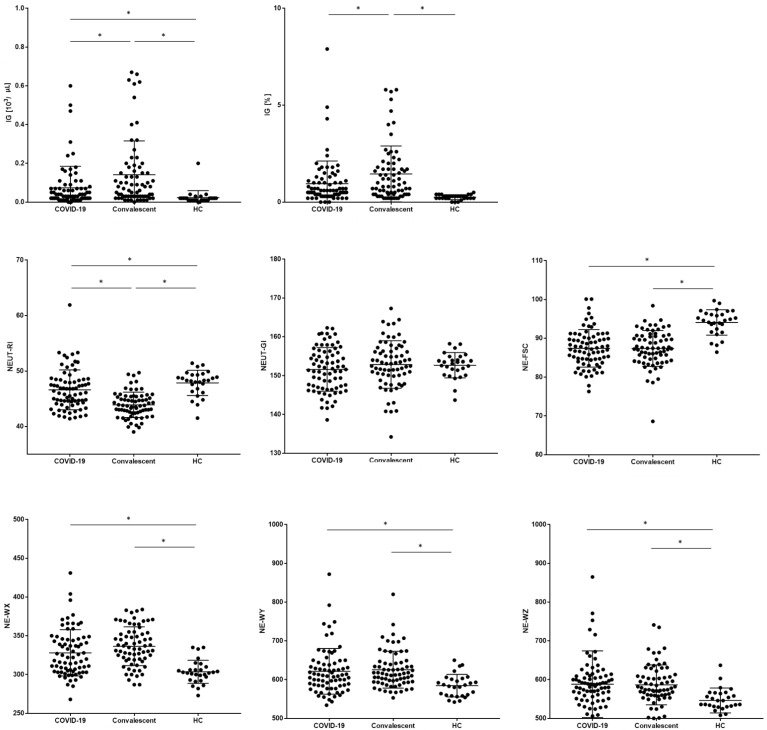
Differences in the proportion of new neutrophil parameters between patients with COVID-19, convalescent, and healthy control (HC) groups. Data expressed as median (Min–Max). * indicates *p* statistically significant.

The proportion of the NEUT-FSC parameter was lower in COVID-19 patients than in HC, and lower in convalescent patients with COVID-19 than in HC. We observed the lowest proportion of NE-WX, NE-WY, and NE-WZ parameters in the HC group (Figure 2, Table 4). We did not observe differences in the proportions of NE-WX, NE-WY, and NE-WZ parameters between COVID-19 patients and convalescent patients with COVID-19.

There were 46 cases in the convalescent group that were the same patients after COVID-19 recovery. The differences in the proportion of Sysmex parameters connected with neutrophils in individual patient with COVID-19 and COVID-19 recovery were presented in Figure S1. The presented differences for 46 patients show the same trends as in the all study group consisting of 79 patients with COVID-19 and 71 convalescents. Therefore, we decided to present results for a larger group in the results section, and additionally show the results for the group *n* = 46 as supplementary data (Table S1).

In addition, we analyzed the correlations between routine morphological parameters and new parameters associated with neutrophils. We observed positive and statistically significant correlations between the number of IG and PLT, which were strongest in COVID-19 patients (r = 0.41, *p* < 0.05) and weaker in convalescents (r = 0.31, *p* < 0.05); this relationship was absent in healthy subjects (Figure S2). We also observed a positive and statistically significant correlation between the IG count and the neutrophil count, which was strongest in COVID-19 patients (r = 0.71, *p* < 0.05) and weaker in convalescents (r = 0.68, *p* < 0.05); this relationship was absent in healthy subjects (Figure S3). Due to the selection of a coherent group, we did not correlate the results with the severity of the disease and the treatment method.

### 3.3. Morphological and New Sysmex Parameters Depending on the Severity of COVID-19

All patients in the study group were at a moderate stage of COVID-19. Thus, we divided the patients according to the presence, or not, of respiratory failure, in addition to oxygen supplementation or not.

We observed that patients with oxygen supplementation had a statistically significantly higher neutrophil percentage and absolute number, and a lower percentage of lymphocytes. Additionally, patients with oxygen supplementation showed a higher absolute number of IG than patients without oxygen supplementation (Table 5).

Additionally, we noticed that patients with respiratory failure showed a higher percentage of NEUT-RI compared to patients without respiratory failure. Interestingly, these patients were not equal to each other in terms of the basic morphological parameters (Table 6).

## 4. Discussion

In our study, we focused on the assessment of selected new neutrophil parameters to characterize convalescent patients and distinguish them from patients with active COVID-19 and healthy volunteers.

Firstly, we characterized the leukocyte subpopulation profile of COVID-19 patients and convalescent patients. We observed leukopenia with an increased proportion of neutrophils (statistically insignificant for neutrophils) in patients with active SARS-CoV-2 infection compared to convalescents, whereas convalescents had a lower neutrophil proportion and elevated PLT levels. We hypothesized that the proportion of neutrophils, in addition to PLT, may be the first indication of recovery in COVID-19 patients. However, solely assessing the level of these cells is not sufficient to distinguish an active infection from an inactive infection and from healthy donors.

### 4.1. Role of Neutrophils in SARS-CoV-2 Infection

Thus, it appears to be justified to measure not only the level of neutrophils, but also to evaluate their functions in the course of infection and during recovery, using new research parameters assessing maturation, reactivity, and granularity. It is known that neutrophils play a key role in the innate first line defense against microbes [31]. Neutrophils phagocytose and kill microorganisms by releasing cytoplasmic granules containing proteases, defensins, antimicrobial peptides, or reactive oxygen species (ROS) [32]. Neutrophils can also form NETs composed of chromatin and neutrophil granular proteins, which are actively released in response to an infection [33]. In the course of COVID-19, elevated levels of NETs were found and an increase in plasma NETs was correlated with the severity of the disease [34,35].

The activity of neutrophil defense mechanisms against microbes may differ depending on the maturity of the cell. The ability to phagocytose and produce ROS increases with cell maturation [36,37], but aging of neutrophils during circulation leads to gradual degranulation that reduces their ability to form NETs [38]. In our study we observed a higher absolute count of IG in convalescent patients than in COVID-19 patients and HC. Silvin A et al. showed an increase in immature neutrophils and noted that the severity of the COVID-19 disease is associated with the number of immature cells. The increase in IG can be a parameter that indicates both an active infection and the healing process [39]. The increased value of immature granulocytes in COVID-19 convalescents may be associated with the deficiency of mature well-functioning neutrophils. Viral infections, sepsis, or other serious injuries can activate the circulating pool of mature neutrophils and, additionally, induce emergency granulopoiesis, which rapidly increases the de novo production of neutrophils [40]. This mechanism results in the presence of both immature neutrophils and mature populations in the peripheral blood, which can act in either an immunosuppressive or pro-inflammatory manner [41,42]. In our study, it was also noted that the pool of immature granulocytes was higher in active COVID-19 than in HC, but it is not as high as in convalescent patients. In patients with active COVID-19, a mature pool of efficient neutrophils can also be expected. Other studies have shown that the pathophysiology of severe COVID-19 is characterized by altered neutrophil abundance, phenotype, and functionality [43,44,45]. Following SARS-CoV-2 infection, an increased number of neutrophils was observed in the nasopharyngeal epithelium [46] and subsequently in the more distal parts of the lungs [47]. Elevated neutrophil counts have also been detected as a clinical feature in the blood of COVID-19 patients [48], and activation of neutrophils is an important feature in severe cases [43,49]. In our study, we hypothesize that a similar absolute neutrophil count and IG count in active COVID-19 and healthy patients results from the selection of the test group. Patients with COVID-19 were in the moderate stage of the disease, and only seven had invasive ventilation. Thus, they may not manifest disease with significant neutrophilia and a high IG count. However, when we tried to distinguish the COVID-19 patients due to the available parameters indicating the severity of the disease, such as oxygen supplementation, we observed that patients with oxygen supplementation had a statistically significantly higher percentage and absolute count of neutrophils and a lower percentage of lymphocytes. In addition, patients with oxygen supplementation had a higher absolute count of IG than patients without oxygen supplementation (Table 5). This observation was consistent with the above literature data of neutrophilia and an increased IG count according to the advancement of the disease in COVID-19 patients.

### 4.2. NEUT-RI

In our study, we noted differences in the parameter indicating neutrophil reactivity, NEUT-RI, between patients with active infection and convalescence patients. Convalescence patients had a significantly lower value of NEUT-RI compared to patients with active infection. Interestingly, in HC, this parameter had the highest value. We hypothesized that in convalescent patients, the lowest proportion of NEUT-RI may be evidence of post-disease depletion of this subpopulation of neutrophils. In the absence of active disease, neutrophils remain inactive and do not show reactivity, and may also be depleted for a relatively short period of time after disease. In contrast, in healthy patients, the increased value of NEUT-RI may indicate a normal neutrophil functionality and the ability of neutrophils to defend against infections. Martens et al. also noted the highest level of this parameter in HC and the lowest in patients with cytokine storms [24]. In other studies Dennison et al. indicated NEUT-RI parameter as an independent predictor for mechanical ventilation and death in COVID-19 patients [25]. Additionally, in our study we noticed that COVID-19 patients with respiratory failure showed a higher percentage of NEUT-RI compared to patients without respiratory failure. Interestingly, these patients were not equal to each other in the basic morphological parameters (Table 6). We hypothesized the NEUT-RI parameter is the only sensitive parameter that, in a consistent moderate COVID-19 group, will help differentiate patients with respiratory failure from patients without respiratory failure. It would be worth examining this parameter at different stages of the disease in the future.

### 4.3. NEUT-GI

Surprisingly, we did not observe differences between the study groups in the value of the functional neutrophil parameter, NEUT-GI. In the study by Schulte-Schrepping J. et al., who evaluated patients with mild and severe COVID-19, functional analysis showed no change in the phagocytic ability of neutrophils [50]. However, ROS production after co-cultivation with Escherichia coli or stimulation with Phorbol-12-myristat-13-acetate was significantly decreased in neutrophils from severe COVID-19 compared to mild patients or controls. Zini G et al. assessed morphological abnormalities of circulating blood cells in COVID-19 patients on smears. In particular, they highlighted the presence of numerous, crowded, dark granulations in the neutrophil cytoplasm (similar to “toxic” granules) and of peripheral light blue agranular areas. In a few cases, the reduction of cytoplasmic granularity was observed. They also noted changes in the shape of the neutrophil nuclei with an increase in band forms and dysmorphic cells with a complete lack of nuclear segmentation [51]. Middleton E. et al. reported lower granularity of neutrophils in COVID-19 patients, as measured by flow cytometry methods [52].

In activated neutrophils there is increased granularity and vacuoles, which result in increased SSC values. In the inflammatory process, there is an accelerated exchange of the pool of circulating neutrophils. This is associated with the appearance of immature and band neutrophils, which have less ability to light scattering, leading to a decrease in SSC value. This may be the reason for the lack of significant differences in NEUT-GI between the study groups [29].

Our observations and the above results suggest that it is not entirely clear whether neutrophils are hypo- or hypergranulated during COVID-19. The discrepancies in the obtained results may be caused by the use of various methods for assessing neutrophil granularity. We suggest that the NEUT-GI parameter is not appropriate for monitoring active COVID-19 and distinguishing these patients from convalescent and HC groups.

### 4.4. NE-FSC

Moreover, we analyzed differences between the study groups for the NE-FSC parameter value. In patients with active COVID-19, the NE-FCS value was lower than in HC, and no differences were seen between patients with active COVID-19 and convalescents. Martens R.J.H. et al. also observed that, in COVID-19 patients, NE-FSC was lower than in the control; in addition, it decreased with the severity of the disease [24]. In the case of other infections, the changes and significance of this parameter were also examined. Buoro S. et al. did not find differences in patients from the intensive care unit caused by sepsis in comparison to HC in the NE-FSC parameter value [53]. Shekhar R. et al. noted a reduction in the size of neutrophils expressed by the NE-FSC parameter in patients with bacterial infection compared to the control group [54]. Lee A.J. et al. showed that the mean volumes of neutrophils were higher in the sepsis group than in the localized infection and control groups [55]. These differences in results are likely due to a different type of infection.

A decrease in the size of a neutrophil could be characteristic for a certain group of infections. In our test group we observed lower NEUT-RI activity and reduced NEUT-GI granularity compared to the control group. Cells appear to be depleted following degranulation, which may explain their reduced size. It is known that infections activate neutrophils and other leukocytes to undergo structural changes that enable them to phagocytose and produce pro-inflammatory cytokines, and this can affect the size of the cells [56].

### 4.5. NE-WX, NE-WY, NE-WZ

NE-WX, NE-WY, and NE-WZ parameters are calculated according to distribution width, and represent the distribution range of the neutrophil population with respect to granularity, activity, and cell volume, excluding outliers below a peak height of 20% in the distribution curve [57].

The neutrophil population data provide information about their morphology and functional activity. They can offer valuable information on the state of neutrophil activation and functional activity. In our study, values of NE-WX, NE-WY, and NE-WZ were higher in COVID-19 patients and convalescents than in HC. It may also suggest that changes in the values of these parameters are an indicator of response for infections. Few studies have assessed the usefulness of these parameters in COVID-19 patients.

Schulte-Schrepping J. et al. found significant differences in NE-WX, NE-WY, and NE-WZ parameters in COVID-19 patients, depending on the severity of the course. Severely ill patients displayed increases in the width of dispersion in terms of activity, granularity, and cell volume compared to patients with a mild course. They confirmed increased cellular heterogeneity, immaturity, and dysregulation of neutrophils in severe COVID-19 [50]. Reports have been published on the utility of neutrophil population parameters in other disease entities. Luo Y et al. found that neutrophil positional parameter values were significantly higher in septic patients. In addition, these parameters correlated with CRP and PCT values [58]. Several studies emphasize the role of these parameters in the assessment of neutrophil dysplasia in myelodysplastic syndromes [59,60]. Some authors point to these parameters as markers that can provide useful information for detecting and monitoring the course of an infection [61,62].

### 4.6. Combining Morphological Parameters with New Neutrophil Parameters

We showed a statistically significant positive correlation between PLT and IG levels, and between the quantity of neutrophils and IG. We showed a similar but weaker correlation in convalescent patients, and no correlation in healthy patients. The presented correlations show that the combination of the assessment of basic morphology parameters such as PLT count, and the absolute number of neutrophils correlated with the amount of IG, allows us to differentiate patients with active COVID-19 from convalescent and, above all, healthy patients.

## 5. Conclusions

We conclude that the newly diagnosed COVID-19 patients should undergo a comprehensive differential analysis of WBC with research parameters for neutrophil activation to identify morphological predictors of recovery or disease progression.

In our study, we showed that recovering patients differ from patients with active disease by showing an increase in the PLT count and subpopulation of leukocytes, i.e., lymphocytes, monocytes, eosinophils, and basophils, and are additionally characterized by an increase in immature granulocyte count and a decrease in reactive granulocytes. Our study implicated that activated neutrophils are an important component in the pathophysiology of SARS-CoV-2 infection, and the NEUT-RI parameter may be an easily accessible and quick indicator of these cells’ activation.

In conclusion, it appears that the use of new neutrophil-related parameters may be of key importance in the recovery process. When combined with basic morphological parameters, monitoring of neutrophils may be crucial to predicting disease outcomes in hospitalized patients and provide a robust element in therapeutic interventions.

## Figures and Tables

**Table 1:** Description and significance of new neutrophil parameters.

Parameters	Parameters Description
**IG** **(Immature Granulocytes)**	The IG fraction includes promyelocytes, myelocytes and metamyelocytes (blasts and band cells are not included).
**NEUT-RI** **(Neutrophil Reactivity Intensity)**	Represents the mean value of fluorescence intensity and increases in proportion to the content of nucleic acids in the cell. It reflects metabolic activity of neutrophils
**NEUT-GI** **(Neutrophil Granularity Intensity)**	Dependent on neutrophil complexity. Increases in the presence of cytoplasmic granulation or vacuoles.
**NE-WX**	Reflects the width of dispersion of neutrophils population, respect to neutrophil side-scatter (NE-SSC).
**NE-WY**	Represents the fluorescence distribution width of neutrophil population, respect to neutrophil fluorescence intensity (NE-SFL).
**NE-WZ**	Reflects the distribution width of neutrophils population, respect to neutrophil forward scatter (NE-FSC).Proportional to the width of dispersion of neutrophil cell size.
**NE-FSC**	Neutrophil forward scatter mean intensity. Reflects neutrophil cell size.

**Table 2:** Demographic and laboratory data of COVID-19 patients.

	Patients *n* = 79
Sex: f/m (*n*)	30/49
Age (mean ± SD years)	58.0 ± 15.0
Women (mean ± SD years)	57.9 ± 14.8
Men (mean ± SD years)	58.4 ± 14.4
Clinical symptoms (*n*, %)	
- Fever	69, 87.3
- Cough	52, 65.8
- Dyspnea	49, 62.0
- respiratory failure	18, 22.7
Diseases comorbidities (*n*, %)	
- diabetes	14, 17.7
- hypertension	30, 38.0
- obesity	11, 13.9
- coronary heart disease	13, 16.5
- neoplastic diseases	7, 8.8
Saturation (mean ± SD years)	91.8 ± 6.1
Chest X-ray changes (*n*, %)	62, 78.5
Oxygen supplementation (*n*, %)	64, 81.0
Invasive ventilation (*n*, %)	7, 8.7

**Table 3:** Proportion of Sysmex morphological parameters in patients: with COVID-19 (A), convalescent patients (B), and control group (HC) (C). Data expressed as median (Q1–Q3). * indicates *p* statistically significant.

Sysmex Parameters Median [(Q1–Q3)]	A. COVID-19 *n* = 79	B. Convalescent Patients *n* = 71	C. HC *n* = 20	* *p* < 0.05 A-B-C ANOVA Kruskal- Wallis	* *p* < 0.05 in Groups Post-Hoc
- WBC [10^3^/µL]	6.51 (4.35–8.73)	7.56 (5.86–9.90)	5.95 (4.78–6.53)	* *p* = 0.0002	A–B, B–C
- NEUTROPHILS [10^3^/µL]	4.02 (2.64–7.04)	4.81 (3.52–6.91)	3.63 (2.36–3.90)	* *p* = 0.0018	B–C
- LYMPHOCYTES [10^3^/µL]	1.00 (0.70–1.47)	1.69 (1.26–2.37)	1.63 (1.29–2.08)	* *p* = 0.0038	A–B, A–C
- MONOCYTES [10^3^/µL]	0.41 (0.26–0.55)	0.70 (0.49–0.83)	0.54 (0.36–0.65)	* *p* < 0.0001	A–B, B–C
- EOSINOPHILS [10^3^/µL]	0.01 (0.00–0.08)	0.10 (0.04–0.17)	0.12 (0.08–0.19)	* *p* < 0.0001	A–B, A–C
- BASOPHILS [10^3^/µL]	0.01 (0.01–0.02)	0.03 (0.02–0.04)	0.04 (0.02–0.04)	* *p* < 0.0001	A–B, A–C
- PLT [10^3^/µL]	227 (184–300)	338 (260–421)	249 (213–278)	* *p* < 0.0001	A–B, B–C
- NEUTROPHILS [%]	74.3 (58.0–85.3)	64.2 (57.3–76.9)	57.2 (52.8–61.8)	* *p* < 0.0001	A–C, B–C
- LYMPHOCYTES [%]	18.1 (9.7–29.0)	24.3 (14.5–31.8)	29.7 (25.8–33.4)	* *p* = 0.0004	A–C, B–C
- MONOCYTES [%]	6.1 (4.2–9.5)	8.7 (7.3–10.1)	8.6 (6.7–9.9)	* *p* = 0.0048	A–B
- EOSINOPHILS [%]	0.1 (0.0–1.4)	1.4 (0.4–2.3)	2.1 (1.2–3.1)	* *p* < 0.0001	A–B, A–C
- BASOPHILS [%]	0.2 (0.1–0.4)	0.3 (0.2–0.5)	0.6 (0.5–0.8)	* *p* < 0.0001	A–B, A–C, B–C

Abbreviations: HC, healthy control; PLT, platelets; WBC, white blood cell count.

**Table 4:** Proportion of Sysmex parameters connected with neutrophils in patients: with COVID-19 A, convalescent patients with COVID-19 B, and control group (HC) C. Data expressed as median (Q1–Q3). * *p* indicates statistically significant.

Sysmex Parameters [Median (Q1–Q3)]	A. COVID-19 *n* = 79	B. Convalescent Patients *n* = 71	C. HC *n* = 20	* *p* < 0.05 A-B-C ANOVA Kruskal- Wallis	* *p* < 0.05 in Group Post-Hoc
- IG [10^3^/µL]	0.03 (0.02–0.07)	0.08 (0.03–0.18)	0.02 (0.01–0.02)	* *p* < 0.0001	A–B, A–C, B–C
- IG [%]	0.6 (0.3–1.1)	0.9 (0.4–1.8)	0.3 (0.2–0.3)	* *p* < 0.0001	A–C, B–C
- NEUT-RI [FI]	46.1 (44.1–48.4)	43.7 (42.5–45.4)	48.3 (46.5–49.3)	* *p* < 0.0001	A–B. A–C, B–C
- NEUT-GI [SI]	151.6 (146.8–156.1)	153.1 (150.1–156.3)	153.3 (150.8–154.4)	*p* = 0.1689	−
- NE-FSC [ch]	87.2 (84.4–90.2)	87.4 (84.6–90.6)	94.6 (92.0–96.2)	* *p* < 0.0001	A–C, B–C
- NE-WX	322 (304–346)	335 (320–354)	302 (295–308)	* *p* < 0.0001	A–C, B–C
- NE-WY	609 (584–642)	616 (594–642)	584 (559–602)	* *p* = 0.0001	A–C, B–C
- NE-WZ	588 (559–610)	582 (561–629)	541 (530–565)	* *p* < 0.0001	A–C, B–C

Abbreviations: ch, channel unity; HC, healthy control; IG, Immature Granulocyte count; NE-FSC, size or volume of neutrophils; NEUT-GI, neutrophil granularity index; NEUT-RI, neutrophil reactive index; NE-WX, reflects the width of dispersion of neutrophils population, with respect to neutrophil side-scatter (NE-SSC); NE-WY, represents the fluorescence distribution width of neutrophil population, with respect to neutrophil fluorescence intensity (NE-SFL); NE-WZ, reflects the distribution width of neutrophils population, with respect to neutrophil forward scatter (NE-FSC); this is proportional to the width of dispersion of neutrophil cell size.

**Table 5:** Proportion of basic morphological Sysmex parameters and new parameters connected with neutrophils in COVID-19 patients with oxygen supplementation and without oxygen supplementation. Data expressed as median (Q1–Q3). A * indicates *p* statistically significant.

Sysmex Parameters [Median (Q1–Q3)]	Oxygen Supplementation *n* = 64	Without Oxygen Supplementation *n* = 15	* *p* < 0.05 The Mann–Whitney U Test
- NEUTROPHILS [103/µL]	4.85 (2.89–7.94)	2.87 (2.18–3.97)	* *p* = 0.0076
- LYMPHOCYTES [103/µL]	0.94 (0.65–1.45)	1.27 (0.97–1.99)	*p* = 0.0796
- MONOCYTES [103/µL]	0.41 (0.26–0.55)	0.36 (0.25–0.53)	*p* = 0.6891
- EOSINOPHILS [103/µL]	0.01 (0.00–0.06)	0.04 (0.00–0.09)	*p* = 0.2179
- BASOPHILS [103/µL]	0.02 (0.01–0.02)	0.01 (0.01–0.02)	*p* = 0.2813
- PLT [103/µL]	242 (187–295)	202 (182–325)	*p* = 0.5804
- NEUTROPHILS [%]	77.0 (60.8–85.9)	61.0 (47.6–74.3)	* *p* = 0.0059
- LYMPHOCYTES [%]	16.2 (8.7–27.5)	30.1 (22.7–35.7)	* *p* = 0.0050
- MONOCYTES [%]	6.0 (3.9–9.4)	7.6 (5.7–10.3)	*p* = 0.2132
- EOSINOPHILS [%]	0.1 (0.0–1.4)	1.0 (0.0–2.9)	*p* = 0.1135
- BASOPHILS [%]	0.2 (0.1–0.4)	0.2 (0.2–0.4)	*p* = 0.9040
- IG [10^3^/µL]	0.04 (0.02–0.08)	0.02 (0.01–0.02)	* *p* = 0.0204
- IG [%]	0.60 (0.40–1.20)	0.45 (0.20–0.60)	*p* = 0.0611
- NEUT-RI [FI]	46.8 (44.3–48.4)	45.0 44.1–47.9)	*p* = 0.5804
- NEUT-GI [SI]	151.6 (146.4–156.1)	151.8 (149.3–155.2)	*p* = 0.5764
- NE-FSC [ch]	46.8 (44.3–48.4)	45.0 (44.1–47.9)	*p* = 0.0533
- NE-WX	325 (303–349)	317 (309–337)	*p* = 0.8349
- NE-WY	613 (587–649)	588 (566–624)	*p* = 0.0511
- NE-WZ	592 (570–614)	553 (535–578)	*p* = 0.0601

Abbreviations: ch, channel unity; IG, Immature Granulocyte count; NE-FSC, size or volume of neutrophils; NEUT-GI, neutrophil granularity index; NEUT-RI, neutrophil reactive index; NE-WX, reflects the width of dispersion of neutrophil population, with respect to neutrophil side-scatter (NE-SSC); NE-WY, represents the fluorescence distribution width of neutrophil population, with respect to neutrophil fluorescence intensity (NE-SFL); NE-WZ, reflects the distribution width of neutrophil population, with respect to neutrophil forward scatter (NE-FSC); this is proportional to the width of dispersion of neutrophil cell size; PLT, platelets.

**Table 6:** Proportion of basic morphological Sysmex parameters and new parameters connected with neutrophils in COVID-19 patients with respiratory failure and without respiratory failure. Data expressed as median (Q1–Q3)). * indicates *p* statistically significant.

Sysmex Parameters [Median (Q1–Q3)]	Respiratory Failure *n* = 18	Without Respiratory Failure *n* = 61	* *p* < 0.05 The Mann–Whitney U Test
- NEUTROPHILS [103/µL]	5.58 (2.64–8.20)	3.90 (2.79–6.81)	*p* = 0.4266
- LYMPHOCYTES [103/µL]	0.79 (0.61–1.22)	1.02 (0.70–1.57)	*p* = 0.1603
- MONOCYTES [103/µL]	0.41 (0.27–0.49)	0.40 (0.25–0.55)	*p* = 0.9216
- EOSINOPHILS [103/µL]	0.00 (0.00–0.09)	0.01 (0.00–0.06)	*p* = 0.4828
- BASOPHILS [103/µL]	0.02 (0.01–0.02)	0.01 (0.01–0.02)	*p* = 0.6141
- PLT [103/µL]	209 (187–295)	229 (182–300)	*p* = 0.7943
- NEUTROPHILS [%]	78.2 (62.5–85.9)	72.9 (57.4–85.2)	*p* = 0.3439
- LYMPHOCYTES [%]	16.1 (8.5–25.7)	20.8 (10.4–31.2)	*p* = 0.3206
- MONOCYTES [%]	6.7 (3.3–9.5)	6.1 (4.6–9.4)	*p* = 0.8757
- EOSINOPHILS [%]	0.0 (0.0–2.0)	0.2 (0.0–1.4)	*p* = 0.2024
- BASOPHILS [%]	0.2 (0.1–0.4)	0.2 (0.1–0.4)	*p* = 0.6305
- IG [10^3^/µL]	0.04 (0.02–0.07)	0.03 (0.02–0.08)	*p* = 0.9508
- IG [%]	0.5 (0.4–1.0)	0.6 (0.3–1.1)	*p* = 0.9213
- NEUT-RI [FI]	47.4 (45.5–51.2)	45.8 (43.8–48.0)	* *p* = 0.0377
- NEUT-GI [SI]	153.2 (147.7–157.4)	151.2 (146.4–155.2)	*p* = 0.2936
- NE-FSC [ch]	86.6 (83.2–89.9)	87.3 (84.6–90.4)	*p* = 0.5099
- NE-WX	344 (303–350)	317 (305–339)	*p* = 0.4251
- NE-WY	625 (605–715)	602 (577–633)	*p* = 0.0519
- NE-WZ	604 (681–622)	579 (556–604)	*p* = 0.0597

Abbreviations: ch, channel unity; IG, Immature Granulocyte count; NE-FSC, size or volume of neutrophils; NEUT-GI, neutrophil granularity index; NEUT-RI, neutrophil reactive index; NE-WX, reflects the width of dispersion of neutrophil population, with respect to neutrophil side-scatter (NE-SSC); NE-WY, represents the fluorescence distribution width of neutrophil population, with respect to neutrophil fluorescence intensity (NE-SFL); NE-WZ, reflects the distribution width of neutrophil population, with respect to neutrophil forward scatter (NE-FSC) It is proportional to the width of dispersion of neutrophil cell size; PLT, platelets.

## Data Availability

Not applicable.

## Supplementary Materials

The following are available online at https://www.mdpi.com/article/10.3390/cells10092332/s1, Table S1: Proportion of Sysmex parameters connected with neutrophils in patients with COVID-19 and convalescent patients (in the convalescent group are the same patient after COVID-19 recovery). Data expressed as median (Q1–Q3). A * was marked *p* statistically significant. Figure S1: The differences in the proportion of Sysmex parameters connected with neutrophils in individual patient with COVID-19 and in the same patient after COVID-19 recovery (*n* = 46). * *p* < 0.05 A * was marked *p* statistically significant. Figure S2: The correlations between proportion of plates counts (PLT) and proportion of immature granulocyte count (IG): A. in patients with COVID-19, B. convalescents patients and C. healthy control. Figure S3: The correlations between proportion of absolute counts of neutrophils and proportion of immature granulocyte count (IG): A. in patients with COVID-19, B. convalescents patients and C. healthy control.
