# Supplementary material for: Neutrophil Maturation, Reactivity and Granularity Research Parameters to Characterize and Differentiate Convalescent Patients from Active SARS-CoV-2 Infection
Source: Cells. 2021 Sep 6;10(9):2332. doi: 10.3390/cells10092332 (PMC8472477; doi:10.3390/cells10092332)
Supplement: Supplementary file 1 [file cells-10-02332-s001.zip › cells-1345782-supplementary.pdf]

Supplementary Materials:

Table S1. Proportion of Sysmex parameters connected with neutrophils in patients with COVID-19 and convalescent patients (in the convalescent group are the same patient after COVID-19 recovery). Data expressed as median (Q1–Q3).). A * was marked P statistically significant.

	Sysmex Parameters [median (Q1–Q3)]	A. COVID-19 *n*=46	B. Convalescent Patients *n* = 46	* $p < 0,05$ The Mann–Whitney U Test
-	IG [10^3/μL]	0.04 (0.02–0.11)	0.08 (0.03–0.20)	*p= 0.0013
-	IG [%]	0.5 (0.3–1.0)	1.0 (0.4–2.3)	p= 0.0567
-	NEUT-RI [FI]	46.8 (44.6–48.4)	44.0 (42.4–45.4)	*p< 0.0001
-	NEUT-GI [SI]	152.4 (146.4–157.0)	153.1 (150.1–156.2)	p= 0.1689
-	NE-FSC [ch]	87.2 (84.6–90.0)	87.4 (85.1–90.5)	p= 0.7581
-	NE-WX	321 (303–344)	338 (322–352)	p= 0.0573
-	NE-WY	608 (585–649)	617 (597–647)	p= 0.2569
-	NE-WZ	589 (570–610)	578 (557–630)	p= 0.6702

Abbreviations: ch, channel unity; IG, Immature Granulocyte count; NE-FSC, size or volume of neutrophils; NEUT-GI, neutrophil granularity index; NEUT-RI, neutrophil reactive index; NE-WX, reflects the width of dispersion of neutrophils population, respect to neutrophil side-scatter (NE-SSC); NE-WY, represents the fluorescence distribution width of neutrophil population, respect to neutrophil fluorescence intensity (NE-SFL); NE-WZ, reflects the distribution width of neutrophils population, respect to neutrophil forward scatter (NE-FSC) It is proportional to the width of dispersion of neutrophil cell size

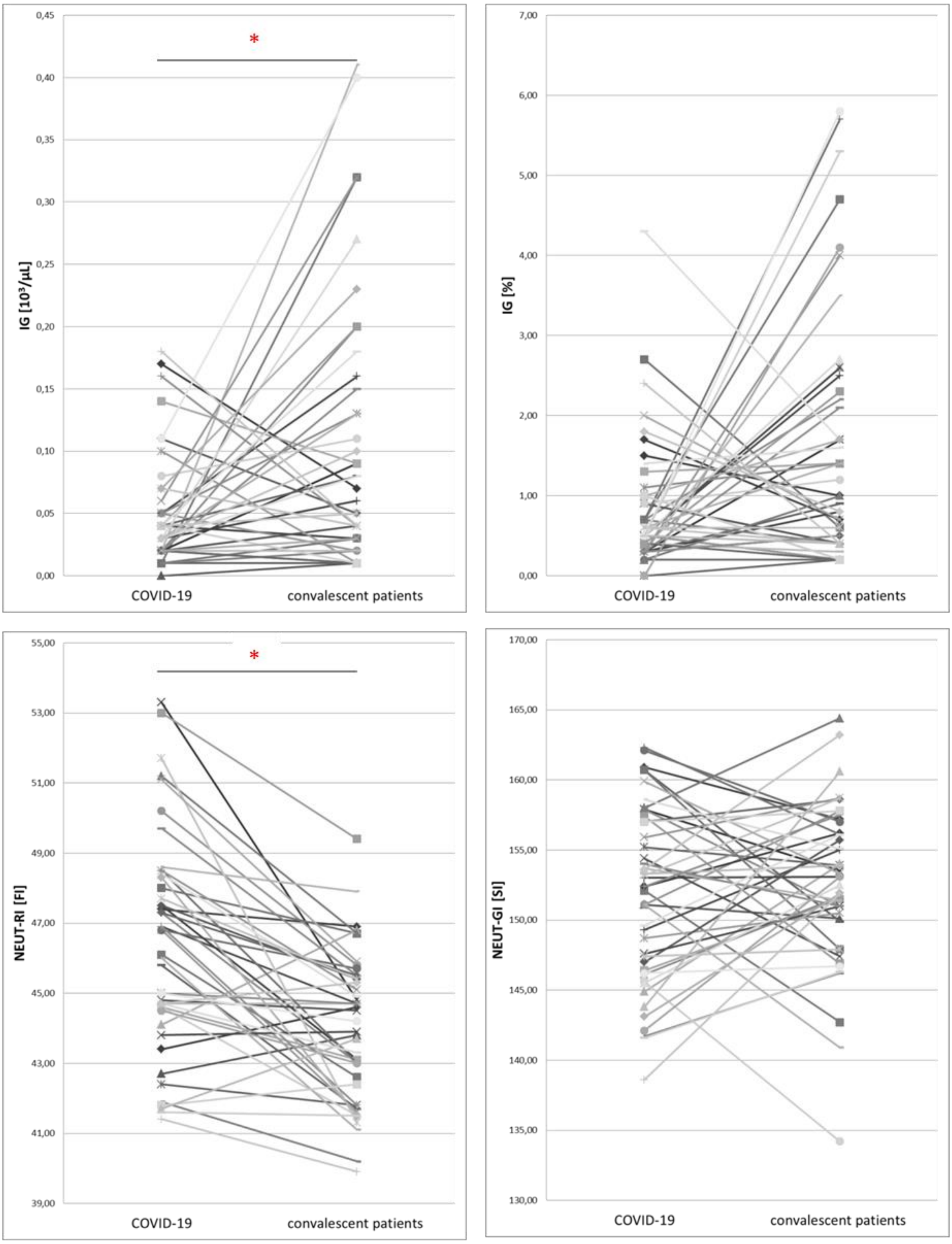

IG [10³/µL]
0,00
0,05
0,10
0,15
0,20
0,25
0,30
0,35
0,40
0,45
*
COVID-19
convalescent patients
IG [%]
0,00
1,00
2,00
3,00
4,00
5,00
6,00
7,00
COVID-19
convalescent patients
NEUT-RI [FI]
39,00
41,00
43,00
45,00
47,00
49,00
51,00
53,00
55,00
*
COVID-19
convalescent patients
NEUT-GI [SI]
130,00
135,00
140,00
145,00
150,00
155,00
160,00
165,00
170,00
COVID-19
convalescent patients

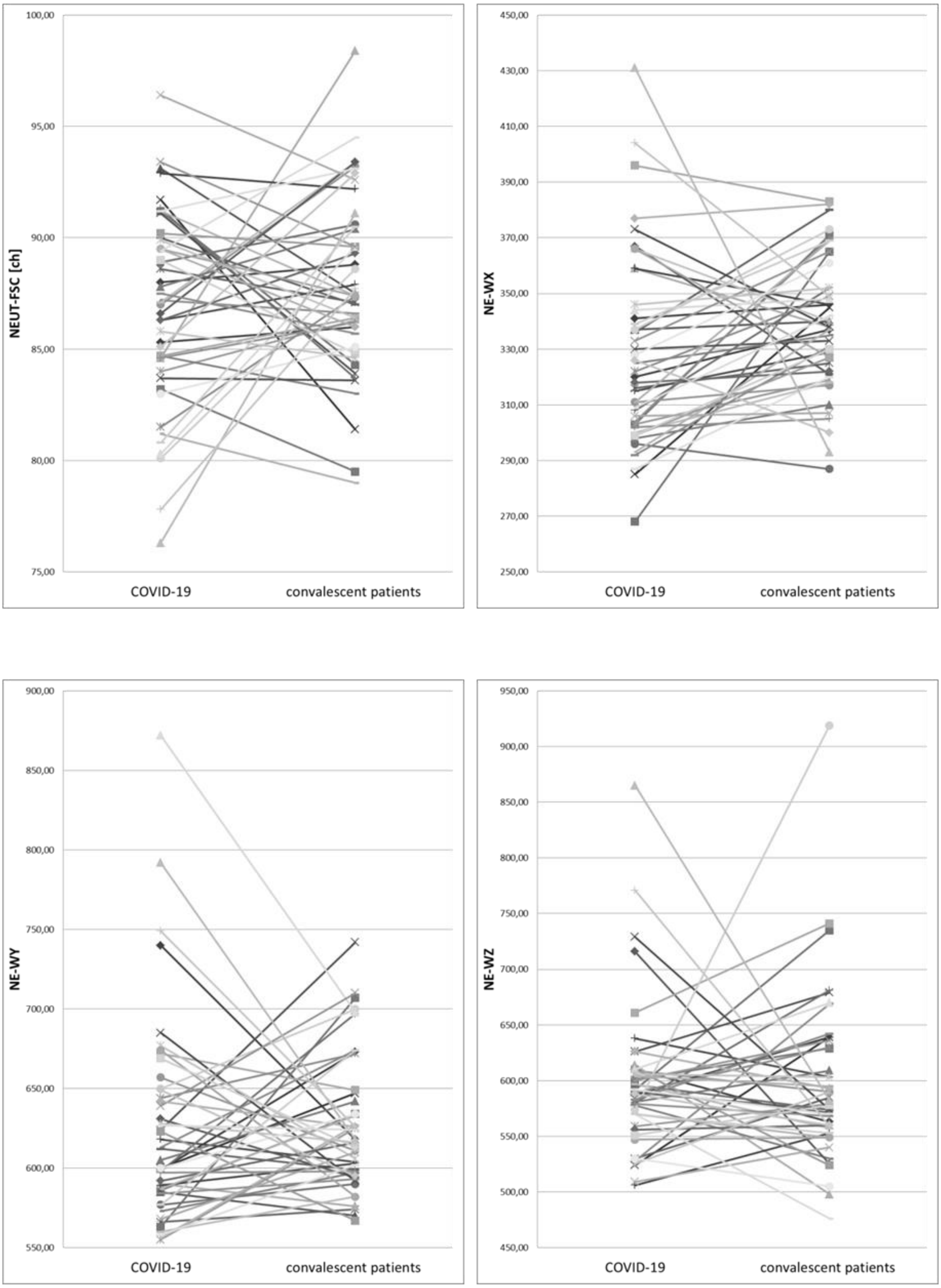

Figure S1. The differences in the proportion of Sysmex parameters connected with neutrophils in individual patient with COVID-19 and in the same patient after COVID-19 recovery (n = 46). A * was marked P statistically significant.

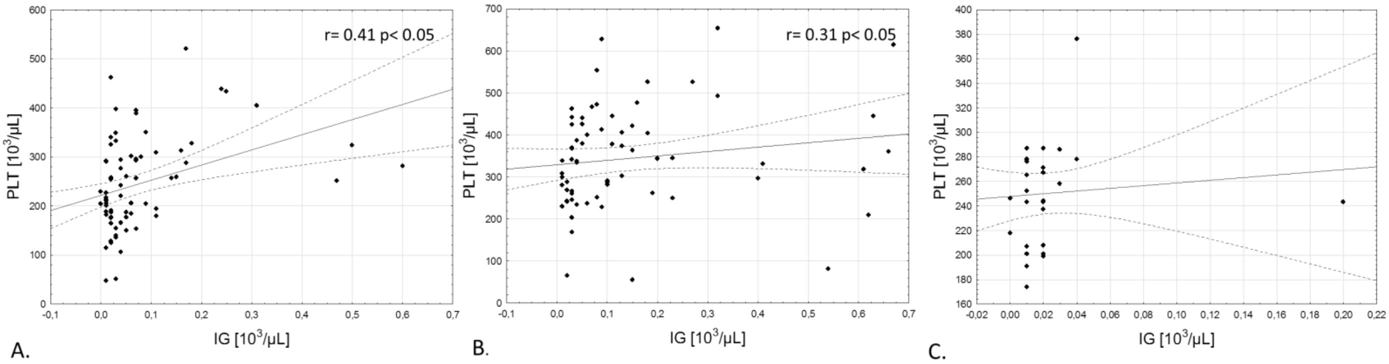

Figure S2. The correlations between proportion of plates counts (PLT) and proportion of immature granulocyte count (IG): **A**. in patients with COVID-19, **B**. convalescent patients and **C**. healthy control.

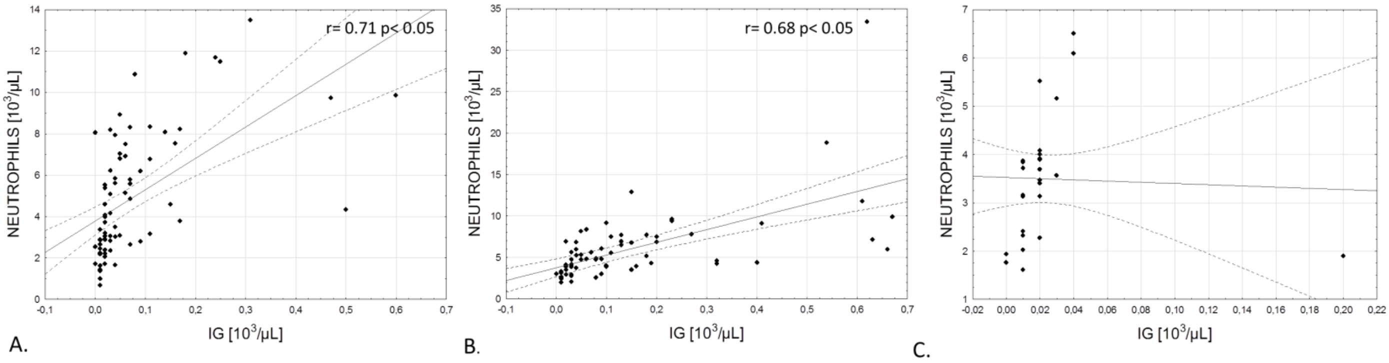

Figure S3. The correlations between proportion of absolute counts of neutrophils and proportion of immature granulocyte count (IG): **A**. in patients with COVID-19, **B**. convalescent patients and C. healthy control.
